# Habitat conditions strongly affect macro- and microelement concentrations in *Taraxacum* microspecies growing on coastal meadows along a soil salinity gradient

**DOI:** 10.7717/peerj.10233

**Published:** 2020-11-24

**Authors:** Beata Bosiacka, Monika Myśliwy, Mateusz Bosiacki

**Affiliations:** 1 Institute of Marine and Environmental Sciences, University of Szczecin, Szczecin, Poland; 2 Department of Functional Diagnostics and Physical Medicine, Faculty of Health Sciences, Pomeranian Medical University in Szczecin, Szczecin, Poland

**Keywords:** Dandelion leaves, Mineral composition, Soil properties, Environmental impact, Soil-plant interaction, Salt marshes

## Abstract

Wild greens can contribute to the human diet as an important source of essential nutrients and drugs. Since environmental factors, including soil properties, may affect the chemical composition of plants, it is necessary among others to assess various habitats in terms of their usefulness for wild plant harvesting and to study impact of environmental factors on the qualitative and quantitative chemical composition of plants. This study was aimed at (1) examining the mineral composition of leaves of three dandelion microspecies, (2) determining the variability of macro- and microelement concentrations in dandelion leaves from populations growing on salty, brackish and non-saline coastal meadows, and (3) assessing the effects of different habitat conditions on the mineral composition of dandelion leaves. It was hypothesized that dandelion microspecies would differ significantly in the mineral composition of leaves. It was also expected that soil conditions would significantly affect nutrient concentrations in dandelion leaves, with soil salinity being the most important factor that differentiated studied populations. Leaves of three dandelion microspecies (*Taraxacum balticum*,* T*. * nordstedtii*, * T*.* haematicum*) were harvested in Baltic costal grasslands, along the soil salinity gradient, to determine macro- and microelement concentrations. Soil samples collected in the closest vicinity of the harvested plants showed the study sites to differ significantly in their soil properties. Moderately saline and organic soils, rich in potassium (K), magnesium (Mg), and calcium (Ca), supported *T*.* balticum*. Moderately or weakly saline and non-saline, organic or mineral soils, with lower median values of K, Mg, and Ca, were typical of *T*.* nordstedtii* sites, while the lowest median values of all the soil properties studied were found for *T*.* haematicum* sites. Our results proved that dandelion microspecies differ significantly in the mineral composition of their leaves. The between-microspecies differences were significant for all the macroelements except magnesium and all the microelements except molybdenum. Most of the macro- and microelements in leaves of the dandelion microspecies correlated positively and significantly with the soil properties, the strongest correlations being found for soil salinity and the leaf Na, Mn, Ca, Fe, K and Zn contents, followed by soil pH and the leaf Na, Mn, Fe, K, Ca, Zn and Mg. Moreover, the impact of soil properties on the mineral contents in leaves of the dandelions we examined seems to be stronger than the genetic differences between dandelion microspecies. Results of our studies on mineral composition of dandelion leaves lend support to the contention that wild greens provide essential mineral nutrients to the diet. Coastal meadows, fed by the brackish water of the Baltic Sea and free of anthropogenic pollution, are a good habitat to collect wild greens from.

## Introduction

With its 2,800 microspecies, the genus *Taraxacum* (Asteraceae, Cichorioideae) is one of the largest and most complicated apomictic complexes (Kirschner et al., 2015). Due to reproductive isolation, agamospermous dandelion microspecies can be constant coexisting taxa (Kirschner & Štěpánek, 1996; Uhlemann, 2001).

The dandelion nomenclature and taxonomic classification raise many doubts. For a long time, dandelions have been treated as the collective species *Taraxacum officinale* (*collectivum, aggregatum*), and the name *T. officinale* is still used to denote the common dandelion in a broad sense (Kirschner & Štěpánek, 2011).

Dandelion microspecies are grouped in about 60 sections. Dandelions of each section occur under a specific set of environmental conditions (Kirschner & Štěpánek, 1996). Certain compounds are common across all the *Taraxacum* sections, but their concentrations vary. Although less than 1% of all the dandelion microspecies have been studied as sources of natural drugs and nutraceutical as well as functional food (Schütz, Carle & Schieber, 2006; Martinez et al., 2015), research tackling dandelion microspecies constituents is growing in importance. Terpenoids and phenolics were isolated from the roots of, e.g., *T. obovatum* (Willd.) DC (Michalska & Kisiel, 2003), *T. udum* Jord. (Michalska, Marciniuk & Kisiel, 2010) and *T. platycarpum* Dahlst. (Warashina, Umehara & Miyase, 2012), whereas aerial parts of, e.g., *T. coreanum* Nakai (Lee et al., 2012), *T. obovatum* (Morales et al., 2014) and *T. mongolicum* Hand-Mazz. (Li et al., 2017) proved a source of flavonoids.

Underground and aerial parts of dandelions have been, for ancient times, used as pharmaceutical raw materials and have been processed into food products or consumed fresh (Schütz, Carle & Schieber, 2006; González-Castejón, Visioli & Rodriguez-Casado, 2012; Martinez et al., 2015). Traditional folk medicine uses, *inter alia*, diuretic and antiedematous properties of dandelions (González-Castejón, Visioli & Rodriguez-Casado, 2012). More recent studies on dandelions have addressed mainly their antioxidant (e.g., Han et al., 2010; Saratale et al., 2018), anti-inflammatory (e.g., Jeon et al., 2008; Koh et al., 2010) and antitumor (e.g., Yamabe et al., 2014; Saratale et al., 2018) properties.

Compared to the growing body of research on the presence of active metabolites in different dandelion organs, the mineral composition and nutritional value of the plant have received much less attention. Just like many other edible wild plants, dandelions can be important as complementary foods. Wild members of the Asteraceae family, including the genus *Taraxacum*, contain higher concentration of minerals, vitamins and essential and fatty acids than many conventional vegetables such as, e.g., lettuce or spinach (Grivetti & Ogle, 2000; Escudero et al., 2003; González-Castejón, Visioli & Rodriguez-Casado, 2012; García-Herrera et al., 2014). All parts of dandelions are edible. These plants can be prepared for human consumption in many ways. Dandelion leaves are usually eaten raw in salads, roasted roots are used as a coffee substitutes, flowers are added to syrups, wines, liquors, desserts etc. (Łuczaj et al., 2012).

Dandelions are typical of open meadow or grassland ecosystems of different degree of moisture. They inhabit also ruderal sites, city lawns, and grow by the roadside. Some dandelion microspecies are tolerant of soil salinity and occur naturally in coastal salt marshes (Dudman & Richards, 1997; Trávníček et al., 2010). Different plant species growing in the same habitat conditions were reported to show different concentrations of nutrients, which can be explained by differences between plants in their absorption of cations from the soil (Shtangeeva, Tesfalidet & Lövgren, 2017). However, it would be necessary to examine whether the differences in concentration of macro- and microelements between plant species growing under different soil conditions will be the same as in the case of plant species growing in the same habitat conditions. It was shown in several studies that nutrient contents in plants are affected e.g., by soil pH and nutrients availability (Rapp et al., 1999; Roca-Pérez, Boluda & Pérez-Bermúdez, 2004). Detailed measurements have to be done in order to explore what habitat conditions exert important and direct influence on macro- and microelements concentrations in leaves (Thompson et al., 1997).

Wild plants used as drugs or food should be harvested in areas free of any pollutants (Kalny et al., 2007). Coastal salt marshes of natural and anthropogenic origin along the Atlantic and Baltic Sea coast in Europe cover up to 176,000 ha (Dijkema, 1990). Coastal salt grasslands are assigned to the EU habitat type 1330 ‘Atlantic salt meadows’ (European Commission, 2013). The habitat is protected under the Natura 2000 network (Habitats Directive 92/43/EEC) and is, in many places, free of pollutants, although threatened by extinction due to, *inter alia*, cessation of traditional management practices (Dijkema, 1990).

Salt marshes along the Baltic Sea coast are affected by seawater of salinity lower than that along the Atlantic coast (the average salinity of the North Sea is 35‰). The Baltic Sea is a brackish sea with a pronounced west-to-east salinity gradient, from 20‰ in the Kattegat to 8.0‰ in the Greifswald Bay to 7.0‰ in the Gulf of Gdańsk to less than 3‰ in the eastern part of the Gulf of Finland (Leppäranta & Myrberg, 2009). However, the seawater salinity is not the only factor affecting soil properties along the sea coast, local conditions being important as well. The suite of the latter includes a limited water exchange, the presence of shallow lagoons, riverine inflow, and meteorological conditions, whereby salinity may be reduced by rainfall or elevated due to evaporation (Hulisz et al., 2013; Hulisz et al., 2016).

Since wild greens can contribute to the human diet as an important source of essential nutrients, it is necessary to assess various habitats in terms of their usefulness for wild plant harvesting, and to study impact of environmental conditions on the qualitative and quantitative chemical composition of plants. This study was aimed at (1) examining the mineral composition of leaves of three dandelion microspecies, (2) determining the variability of macro- and microelement concentrations in dandelion leaves from populations growing on salty, brackish and non-saline coastal meadows, and (3) assessing the effects of different habitat conditions on the mineral composition of dandelion leaves. It was hypothesized that dandelion microspecies would differ significantly in the mineral composition of leaves. Moreover, it was expected that soil conditions would significantly affect nutrient concentrations in dandelion leaves, with soil salinity being the most important factor that differentiated studied populations.

## Materials and Methods

Dandelion leaves were harvested in May 2017 in coastal grasslands of the Wolin Island (NW Poland), within the Natura 2000 area PLH320018 (‘Ujście Odry i Zalew Szczeciński’). The area is amenable to management, including harvesting of plant species that are not protected by law. The central part of the coastal meadows where the collections were made has the following coordinates: N53°54′26.3″–E14°39′14.0″

A total of 40 dandelion leaf samples were collected: 10 samples contained leaves of *Taraxacum balticum* Dahlst. (from salt and brackish meadows); 20 samples consisted of leaves of *T. nordstedtii* Dahlst. (10 from brackish meadows, 10 from non-salty meadows); and 10 samples contained leaves of *T. haematicum* G.E. Haglund ex H. Øllg. & Wittzell (from non-salty meadows). The actual sampling sites were determined, along the soil salinity gradient, based on earlier studies on the distribution of *Taraxacum* microspecies along soil property gradients of meadows at the Polish Baltic Sea coast (Bosiacka et al., 2019). Each dandelion individual yielded 2–3 leaves. Specimens of the dandelion microspecies investigated from the Wolin Island were collected as vouchers and were deposited at the University of Szczecin herbarium (SZUB).

*T. balticum* is one of the most halophilous dandelions, its distribution range being closely associated with the Baltic Sea coast (Kirschner & Štěpánek, 1996). Like other taxa of the section *Palustria*, it is becoming less common, also in Poland (Bosiacka et al., 2016; Bosiacka et al., 2019). *T. nordstedtii* is a quite common microspecies in the western part of the Polish Baltic Sea coast (Bosiacka et al., 2019), and occurs both in typically halophytic phytocoenoses and along their edges, among glycophytes. Compared to other species of the section *Celtica*, the microspecies has the widest ecological spectrum (Sterk, Groenhart & Mooren, 1983; Kirschner & Štěpánek, 1984; Horn et al., 1996). *T. haematicum* belongs to the section *Taraxacum* (*Ruderalia*), commonly found on wet and regularly fertilized grasslands and in various anthropogenic habitats; it is often present in areas affected by the maritime climate (Sterk, Groenhart & Mooren, 1983; Trávníček et al., 2010; Bosiacka et al., 2019). The main diagnostic features of studied dandelion microspecies were summarized in Table 1.

**Table 1:** The main diagnostic features of *Taraxacum balticum, T. nordstedtii* and *T. haematicum* (according to Kirschner & Štěpánek, 1998; Kirschner & Štěpánek, 1984; Ollgaard & Wittzell, 1995, respectively).

	*T. balticum*	*T. nordstedtii*	*T. haematicum*
Size	Small, medium-sized or subrobust	Medium-sized to robust	Rather small to medium-sized
Leaves	Deep green, glabrous; deeply divided, lateral lobes 3-5, linear or narrowly triangular; terminal lobe tripartite, terminal segment lingulate; interlobes entire or with distinct acute linear lobules and filiform teeth	Light green to medium green, usually glabrous; the vernal leaves often not lobed or shallowly lobed, leaf lobes 2–3, almost obtuse, patent and entire; the later leaves usually deeply lobed, lateral lobes 2–4; terminal lobe obtuse at the apex, widely triangular; interlobes wide short, entire or with irregular remote patent acute teeth	Yellowish to somewhat bluish green, glabrous or sparsely arachnoid at the base; lateral lobes patent to somewhat recurved, usually more or less falcate; terminal lobe nor larger than lateral lobes, with a gradual elongate or lingulate apical lobule; interlobes acute angled to widely rounded
Outer bracts	9–13, adpressed, broadly ovate to ovate-lanceolate 6.0–7.5 mm long and 3.5–4.0 mm wide, borders indistinct 0.6-1.0 mm wide	15–20, adpressed to erect, ovate-lanceolate to lanceolate 8.0–9.0 mm long and 2.5–4.0 mm wide, greyish glaucous and green, on upper half purplish bordered, often ciliate	14–17, more or less horizontal, rather regularly arranged, broadly lanceolate, margin flat, with distinct reddish border
Outer ligule striped	grey-green purple	brownish red or brownish purple	usually dark reddish
Stigmas	Greyish yellow	Yellowish to greenish light grey	Discoloured
Pollen	Absent or very sparsely present	Absent	Present, with grains of varying size
Chromosome number	2n = 32	2n = 48	2n = 24
Mode of reproduction	Apomicts	Apomicts	Apomicts

Three soil samples from the plant root zone (0–25 cm) were collected with Egner’s soil sampler in the closest vicinity of each plant from which the leaves were picked off. The three samples were combined to form a single sample to be used in laboratory analyses.

The soil samples were dried at room temperature and sieved to remove particles larger than 1 mm. The sieved material was used to determine the following properties: organic matter content (as loss on ignition at 550 °C); pH (potentiometrically in 1 M KCl); contents of available forms of potassium, magnesium and calcium (in 0.5 M HCl, using the Egner-Riehm method, Schachtschabels method and the method of atomic absorption spectrophotometry, according to the American Society of Agronomy technique, respectively); and electrolytic conductivity of the saturated soil extract (ECe) (conductometrically). Soil salinity ranges were classified according to the following scale: non-saline soils (0–2 dS m^−1^); slightly saline soils (2–4 dS m^−1^); moderately saline soils (4–8 dS m^−1^); strongly saline soils (8–16 dS m^−1^); very strongly saline soils (>16 dS m^−1^) (Richards, 1954).

All leaf samples were stored at −80 °C until analysis. Na, K, Ca, Mg, Cu, Fe, Mn, Zn, Cr, and Mo levels were determined using inductively coupled plasma optical emission spectrometry (ICP-OES, ICAP 7400 Duo, Thermo Scientific) equipped with a concentric nebulizer and cyclonic spray chamber. Analyses were conducted in radial and axial modes. The samples were dried at 90 °C to constant weight, pulverized in a porcelain mortar, and digested using the MARS 5, CEM microwave digestion system. The samples (minimum 0.1 g each) were placed in clean polypropylene vials to which 3 mL of 65% HNO_3_ (Suprapur, Merck) was added; each sample was allowed 30 min pre-reaction time in the clean hood. After the passage of the pre-reaction time, 1 mL of non-stabilized 30% H_2_O_2_ solution (Suprapur, Merck) was added to each vial. Then, the samples in Teflon vessels were heated in the microwaved digestion system for 35 min at 180 °C (15 min ramp to 180 °C and kept at 180 °C for 20 min). Following the digestion, the samples were taken from the microwave and cooled to room temperature. The samples were placed in acid-washed 15 mL polypropylene sample tubes in the clean hood. Then, they were diluted 40 times to perform ICP-OES measurement, and 250 µL was taken from each digest. An internal standard was used to spike the samples and provide a final concentration of 0.5 mg/L Ytrium, one mL of 1% Triton (Triton X-100, Sigma). Finally, the samples were diluted to the volume of 10 mL with 0.075% nitric acid (Suprapur, Merck). Then, the samples were kept in a monitored refrigerator at 8 °C until analysis. An addition of concentrated nitric acid (200 µL) to tubes without samples and subsequent dilution using the aforementioned method were used to obtain blank samples. Multi-element calibration standards (ICP multi-element standard solution IV, Merck) for Na, K, Ca, Mg, Cu, Fe, Mn, Zn, Cr, and Mo were prepared using various concentrations of inorganic elements in the same manner as in blanks and samples. All solutions were made with deionized water (Direct Q UV, Millipore, approximately 18.0 MΩ). The wavelengths (nm) were: 396.847 for Ca, 283.563 for Cr, 324.754 for Cu, 259.940 for Fe, 766.490 for K, 285.213 for Mg, 260.569 for Mn, 589.592 for Na, 213.856 for Zn and 204.598 for Mo.

The basic statistical metrics of macro- and microelement concentrations in dandelion leaves (interquartile ranges of values, medians, outlier values, extreme values) were calculated and illustrated by box and whiskers plots. As the distributions of values deviated from normality, statistical significance of differences was assessed with the Kruskal-Wallis test and the *post-hoc* Dunn’s multiple comparisons test. The relationships between element contents in *Taraxacum* microspecies leaves and soil properties were examined using Spearman’s rank correlation test. Due to a long environmental gradient shown by the results of the Detrended Correspondence Analysis (DCA) (Jongman, ter Braak & van Tongeren, 1995), the Canonical Correspondence Analysis (CCA) was applied to relate the variability of nutrient concentrations in dandelion leaves to soil properties. The Monte Carlo permutation test with 499 unrestricted permutations and the forward selection of environmental variables was used to determine the importance and statistical significance of variables in explaining the variability in mineral composition of leaves. Finally the hierarchical cluster analysis based on the nearest neighbor method was run with the matrix of the population’s mean values. All the analyses were performed with STATISTICA v.12 (Inc, 2013), Canoco v.4.5 (ter Braak & Šmilauer, 2002), and MVSP 3.2 (Kovach, 2010).

## Results

**Figure 1:**
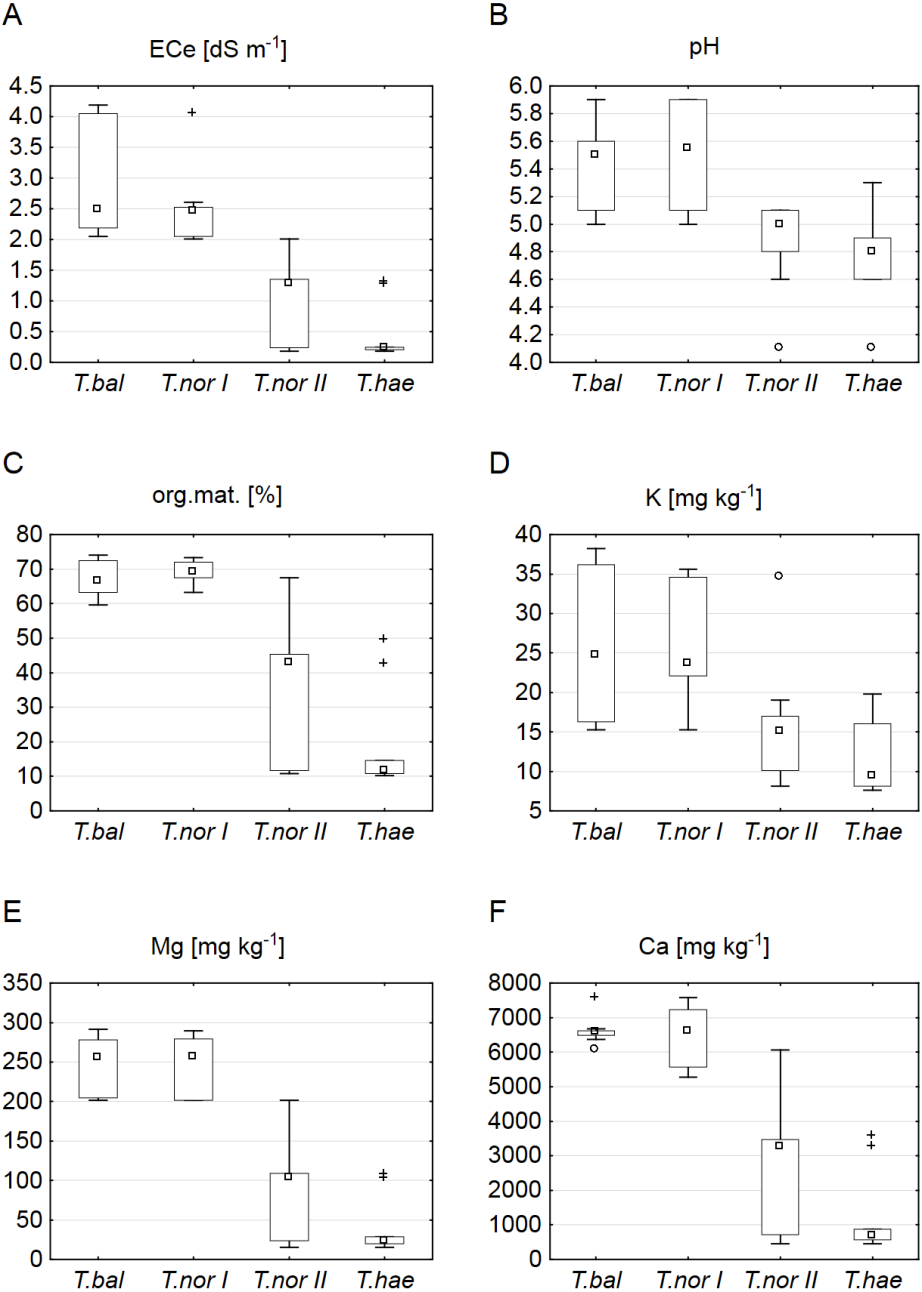
Soil properties related to individual *Taraxacum* microspecies: *T*.* balticum* (*T.bal*), *T*. *nordstedtii* (*T.nor I*, *T.nor II*), and *T. haematicum* (*T.hae*). (A) Electrolytic conductivity of the saturated soil extract (ECe); (B) pH; (C) organic matter content; (D) potassium content; (E) magnesium content; (F) calcium content. Large boxes indicate 25–75% of the interquartile range of values; small boxes represent medians, white circles are outlier values, and crosses represent extreme values.

The sites of dandelion leaf collection were found to differ significantly in their soil properties (Table 2). The soils at sites supporting *Taraxacum balticum* and *T. nordstedtii I* were saline (ECe of 2.05–4.19 and 2.01–4.05 dS m^−1^, respectively) and organic (organic matter contents of 61.3–74.0 and 63.2–73.2%, respectively), least acidic (pH of 5.0–5.8 and 5.0–5.9, respectively), richest in potassium (15.3–38.2 and 15.3–35.6 mg kg^−1^, respectively), magnesium (201.2–291.4 and 201.3–289.4 mg kg^−1^, respectively) and calcium (6,072.0–7,567.8 and 5,272.0–7,577.8 mg kg^−1^, respectively). The soils which supported *T. nordstedtii II* were non-saline (ECe of 0.18–1.98 dS m^−1^), organic or mineral (organic matter content of 10.7–67.5%), with all the other median values of soil parameters being clearly lower (Fig. 1). The lowest median values of all the soil properties studied were typical of the *T. haematicum* site. The soils there were non-saline and mostly mineral, most acidic, poorest in potassium (7.6–19.8 mg kg^−1^), magnesium (15.1–107.9 mg kg^−1^) and calcium (458.0–3,572.0 mg kg^−1^).

**Table 2:** Results of the Kruskal–Wallis and post hoc Dunn’s tests, showing significance of differences in soil parameters between the sites supporting *Taraxacum balticum*, *T. nordstedtii* and *T. haematicum*.

Soil parameter	Kruskal–Wallis test		Dunn’s multiple comparisons test					
			*T.bal-T.nor I*	*T.bal-T.nor II*	*T.bal-T.hae*	*T.nor I-T.nor II*	*T.nor I-T.hae*	*T.nor II-T.hae*
	H	*p*-value	*p*-value	*p*-value	*p*-value	*p*-value	*p*-value	*p*-value
ECe [dS m^−1^]	30.20264	^*^0.0000	1.000000	^*^0.001240	^*^0.000036	^*^0.011294	^*^0.000529	1.000000
pH	22.49743	^*^0.0001	1.000000	^*^0.027034	^*^0.001802	^*^0.019339	^*^0.001194	1.000000
org. mat. [%]	27.87957	^*^0.0000	1.000000	^*^0.018177	^*^0.000488	^*^0.004569	^*^0.000085	1.000000
K [mg kg^−1^]	20.26951	^*^0.0001	1.000000	^*^0.048419	^*^0.002503	^*^0.040796	^*^0.002012	1.000000
Mg [mg kg^−1^]	29.45481	^*^0.0000	1.000000	^*^0.003333	^*^0.000115	^*^0.005812	^*^0.000225	1.000000
Ca [mg kg^−1^]	28.93086	^*^0.0000	1.000000	^*^0.005245	^*^0.000225	^*^0.004116	^*^0.000168	1.000000

**Notes.**

* *p* < 0.05 (significance level).

*T.bal* *Taraxacum balticum* *T.nor I* and *T.nor II* *T. nordstedtii* *T.hae* *T. haematicum*

**Figure 2:**
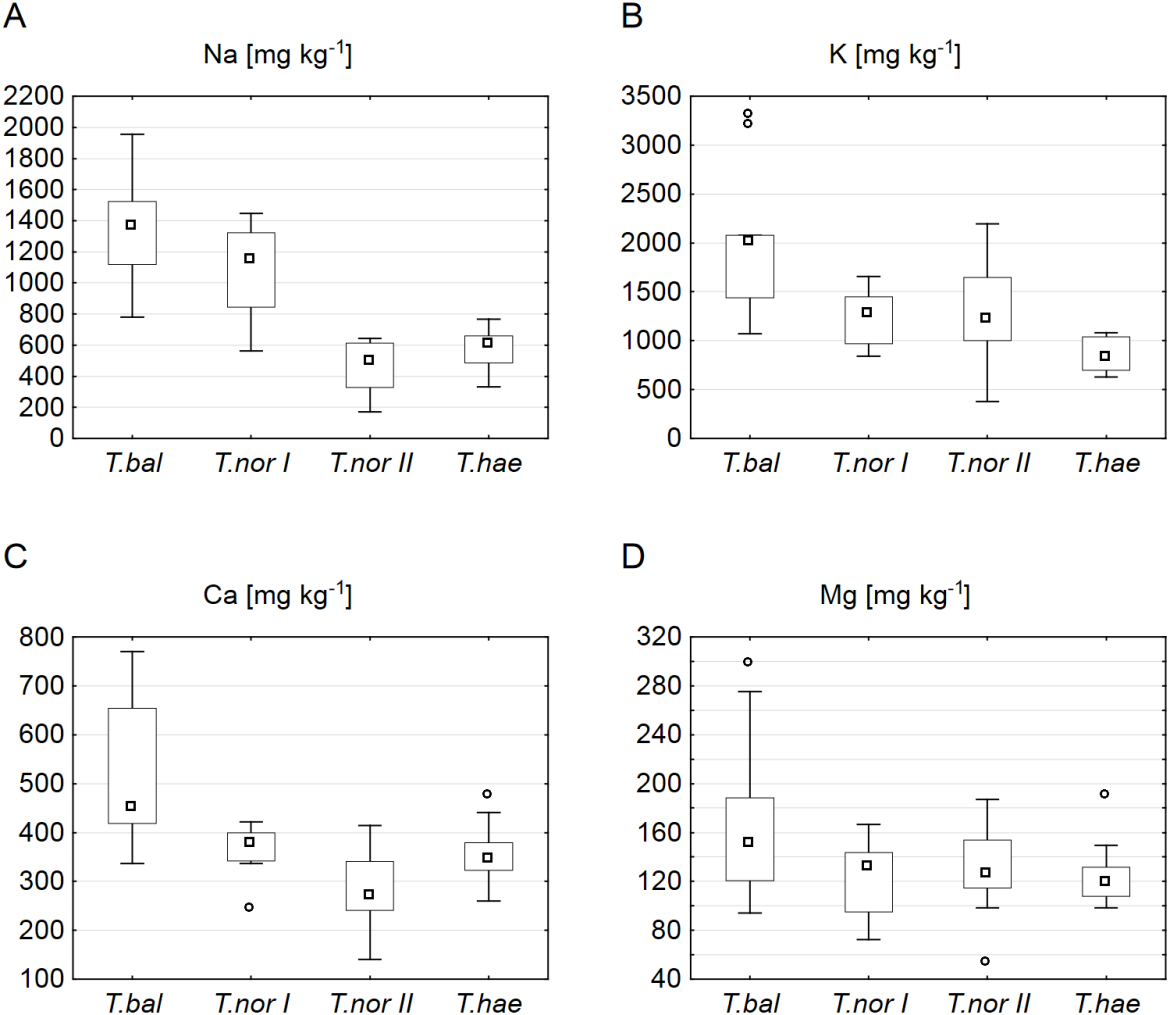
Contents of macroelements in leaves of *Taraxacum balticum* (*T.bal*), *T. nordstedtii* (*T.nor I*, *T.nor II*) and *T. haematicum* (*T.hae*). (A) Sodium (Na); (B) potassium (K); (C) calcium (Ca); (D) magnesium (Mg). Large boxes indicate 25–75% of the interquartile range of values; small boxes represent medians, and white circles are outlier values.

Our analysis of the macroelement contents in dandelion leaves showed the median sodium, potassium, calcium and magnesium contents (1,366.6, 2,006.5, 451.0 and 151.5 mg kg^−1^ dry weight, respectively) to be at their highest in *Taraxacum balticum*. The lowest median sodium and calcium contents (494.2 and 270.3 mg kg^−1^, respectively) were recorded in *T. nordstedtii II*, while the lowest potassium and magnesium contents (827.3 and 119.0 mg kg^−1^, respectively) were found in *T. haematicum* (Fig. 2). The between-microspecies differences were significant for all the macroelements except magnesium (Table 3). In addition, *T. nordstedtii I* differed significantly from *T. nordstedtii II* in terms of the sodium content.

**Table 3:** Results of the Kruskal–Wallis and Dunn’s tests, showing significance of differences in macro- and microelement contents [mg kg^−1^] between leaves of three *Taraxacum* microspecies.

Macro- and microelement	Kruskal–Wallis test		Dunn’s multiple comparisons test					
			*T.bal-T.nor I*	*T.bal-T.nor II*	*T.bal-T.hae*	*T.nor I-T.nor II*	*T.nor I-T.hae*	*T.nor II-T.hae*
	H	*p*-value	*p*-value	*p*-value	*p*-value	*p*-value	*p*-value	*p*-value
Na	28.48829	^*^0.0000	1.000000	^*^0.000020	^*^0.001150	^*^0.002414	0.052690	1.000000
K	19.60244	^*^0.0002	0.175317	0.292953	^*^0.000060	1.000000	0.151368	0.086121
Ca	17.95756	^*^0.0004	0.292953	^*^0.000168	0.062253	0.159015	1.000000	0.623927
Mg	3.026341	0.3876	0.941672	1.000000	0.700673	1.000000	1.000000	1.000000
Cu	26.51854	^*^0.0000	0.292953	^*^0.012441	1.000000	^*^0.000003	0.532161	^*^0.004897
Fe	26.53610	^*^0.0000	0.975758	^*^0.001939	1.000000	^*^0.000004	0.212269	^*^0.023246
Mn	24.17561	^*^0.0000	^*^0.027856	^*^0.001441	^*^0.000018	1.000000	0.397950	1.000000
Zn	22.58634	^*^0.0000	0.306353	^*^0.039636	1.000000	^*^0.000018	0.700673	^*^0.011665
Cr	24.79902	^*^0.0000	0.077393	0.095705	1.000000	^*^0.000006	0.511007	^*^0.008986
Mo	0.444841	0.9308	1.000000	1.000000	1.000000	1.000000	1.000000	1.000000

**Notes.**

* *p* < 0.05 (significance level).

*T.bal* *Taraxacum balticum* *T.nor I* and *T.nor II* *T. nordstedtii* *T.hae* *T. haematicum*

**Figure 3:**
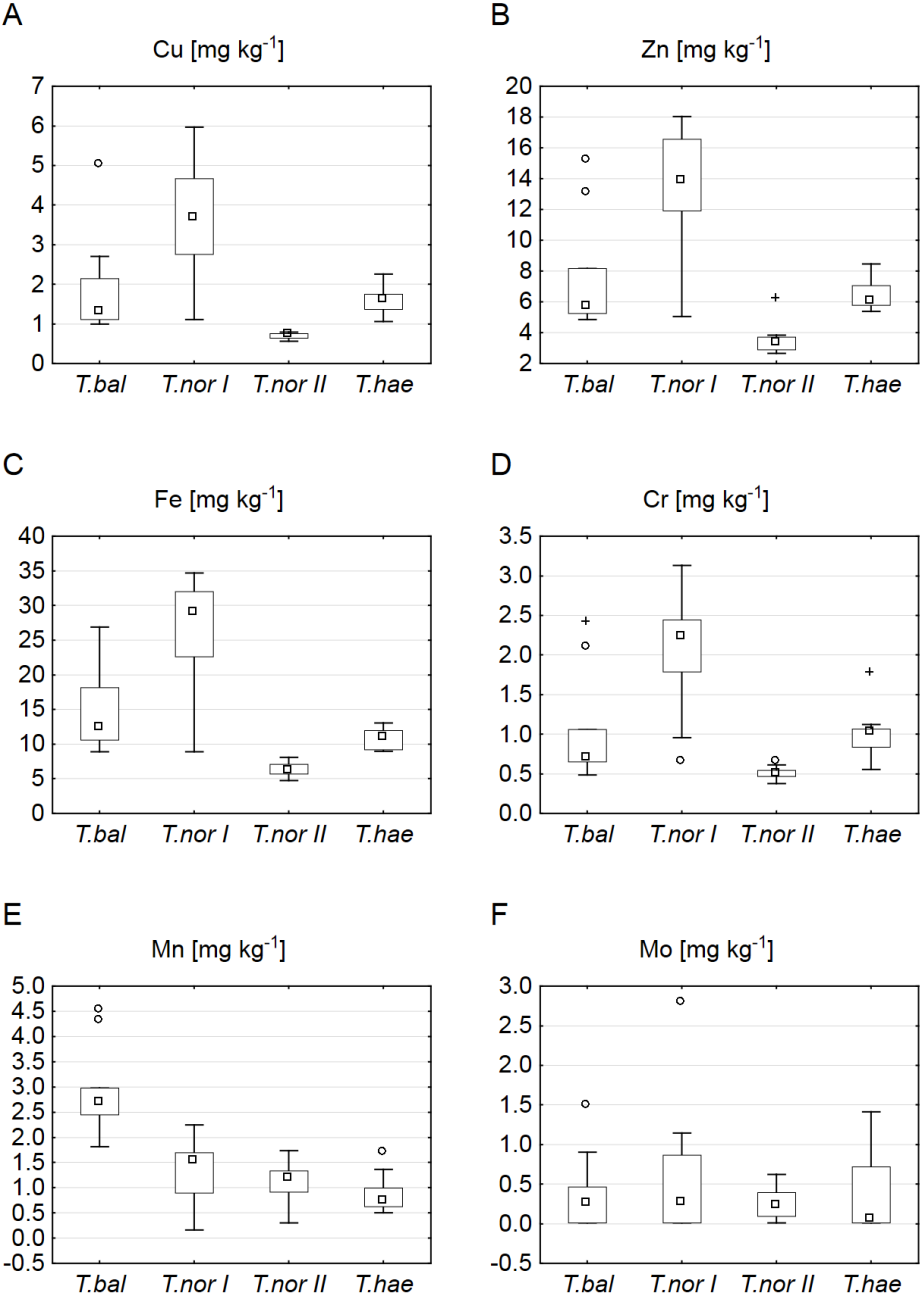
Contents of microelements in leaves of *Taraxacum balticum* (*T. bal*), *T. nordstedtii* (*T.nor I*, *T.nor II*) and *T. haematicum* (*T.hae*). (A) Copper (Cu); (B) zinc (Zn); (C) iron (Fe); (D) chromium (Cr); (E) manganese (Mn); (F) molybdenum (Mo). Large boxes indicate 25–75% of the interquartile range of values; small boxes represent medians, white circles are outlier values, and crosses represent extreme values.

Our analysis of microelements revealed the highest median concentrations to be typical of *T. nordstedtii I*: the median copper, iron, zinc, chromium and molybdenum concentrations were 3.7, 29.0, 13.9, 2.2 and 0.3 mg kg^−1^, respectively. The only exception was manganese, its median concentration being highest (2.7 mg kg^−1^) in *T. balticum*. The lowest median contents of copper, iron, zinc, and chromium were found in *T. nordstedtii II* (0.7, 6.2, 3.4, 0.5 mg kg^−1^, respectively), while the lowest manganese and molybdenum contents were recorded in *T. haematicum* (0.7 and 0.06 mg kg^−1^, respectively) (Fig. 3). The differences in microelement contents between *T. balticum* and *T. nordstedtii II* were significant, except for chromium and molybdenum; also, the differences between *T. nordstedtii I* and *T. nordstedtii II*, as well as those between *T. nordstedtii II* and *T. haematicum* were significant, except for manganese and molybdenum (Table 3).

Correlations between most macro- and microelement contents in leaves of the *Taraxacum* microspecies and the soil properties studied were positive and significant (Table 4), with the exception of molybdenum concentration which did not correlate with any soil parameter. The strongest positive correlations were those between soil salinity and the leaf macro- and microelement contents (particularly Na, Mn, Ca, Fe, K and Zn), followed by soil pH (particularly with Na, Mn, Fe, K, Ca, Zn and Mg) (Table 4).

**Figure 4:**
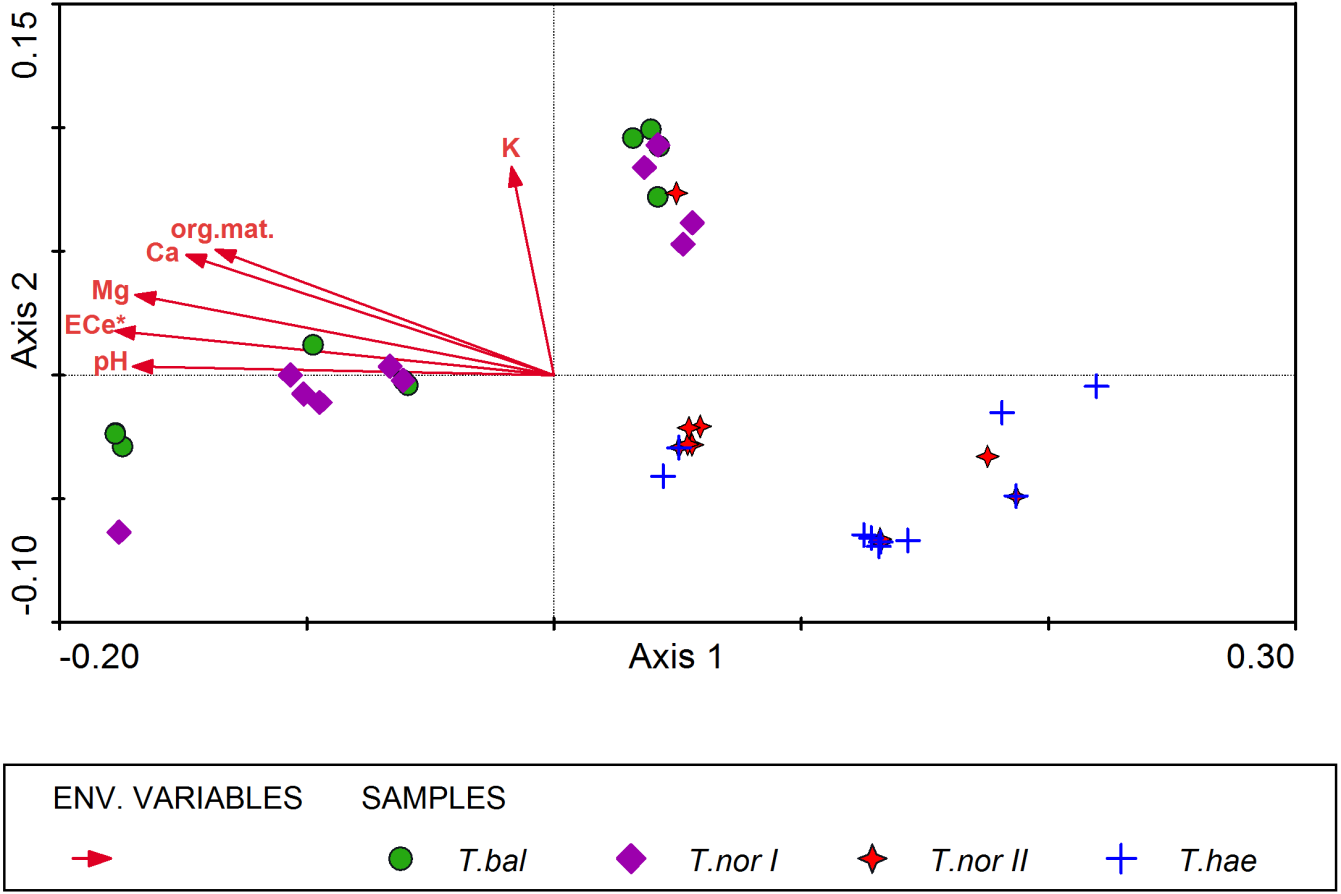
Ordination diagram of *Taraxacum balticum* (*T.bal*), *T. nordstedtii* (*T.nor I*, *T.nor II*) and *T. haematicum* (*T.hae*) samples (colorful symbols), and soil properties (red arrows) along the first two CCA axes. Eigenvalues of Axis I and Axis II: 0.012 and 0.003 respectively; sum of all eigenvalues (total inertia): 0.050; sum of all canonical eigenvalues: 0.016; * denotes statistically significant variable.

All soil parameters included in the CCA explained 32% of total variation in the mineral composition of dandelion leaves. The results of step-wise forward selection of environmental variables showed that soil salinity (ECe) was statistically significant in differentiation of the studied populations of *Taraxacum* microspecies in terms of mineral composition of their leaves (Fig. 4). This variable has also the highest correlation (*r* =  − 0.615) with the sample position along the gradient represented by Axis I (inter-set correlation), followed by soil pH and magnesium content in the soil. On the other hand, potassium content in the soil was most closely correlated with Axis II (*r* = 0.431).

The distribution of samples in the resultant CCA diagram showed three main clusters. Two of them were located in the left-hand part and in the upper part of the diagram and represented *Taraxacum balticum* and *T. nordstedtii I*. The samples located in the left-hand side of the ordination space were associated with the highest values of ECe, soil pH, organic matter content, and the highest contents of magnesium and calcium in the soil. In turn, samples located in the upper part of the diagram were associated with relatively lower values of the soil properties mentioned above, while with the highest potassium content in the soil. The third cluster of samples occupied the lower right part of the diagram and represented *T. haematicum* and *T. nordstedtii II.* They were associated with the lowest values of all soil variables.

**Table 4:** Results of Spearman’s rank correlation test between macro- and microelement contents [mg kg^−1^] in leaves of *Taraxacum* microspecies and soil parameters.

Macro- and microelement	ECe [dS m^−1^]	pH	org. mat. [%]	K [mg kg^−1^]	Mg [mg kg^−1^]	Ca [mg kg^−1^]
Na	^*^0.926573	^*^0.853199	^*^0.710596	^*^0.460997	^*^0.894765	^*^0.838810
K	^*^0.604789	^*^0.519524	^*^0.587451	^*^0.533133	^*^0.582578	^*^0.552291
Ca	^*^0.661409	^*^0.605212	^*^0.495961	^*^0.329592	^*^0.637709	^*^0.576042
Mg	^*^0.472113	^*^0.505431	0.262164	0.036350	^*^0.451092	^*^0.327920
Cu	^*^0.466949	^*^0.476396	^*^0.375540	0.171042	^*^0.493543	^*^0.494649
Fe	^*^0.642911	^*^0.613725	^*^0.489668	0.279998	^*^0.655741	^*^0.609369
Mn	^*^0.722911	^*^0.656474	^*^0.610652	^*^0.443432	^*^0.690116	^*^0.676117
Zn	^*^0.501690	^*^0.506377	^*^0.354594	0.139576	^*^0.515896	^*^0.477094
Cr	^*^0.409014	^*^0.398841	0.303119	0.163528	^*^0.414276	^*^0.408656
Mo	0.085795	−0.058337	0.027649	0.091535	0.039836	0.116626

**Notes.**

* *p* < 0.05 (significance level).

strong correlations are marked in bold.

**Figure 5:**
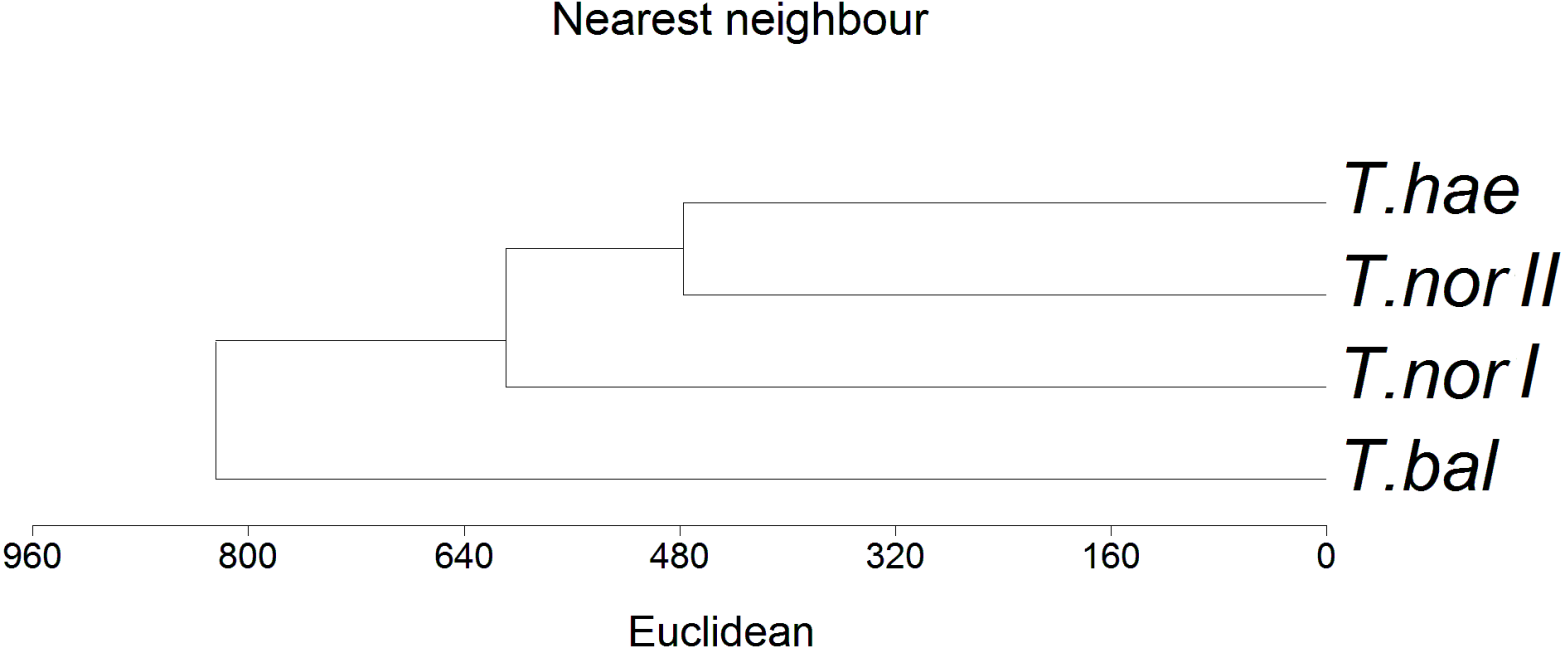
Dendrogram of hierarchical cluster analysis (nearest neighbour method) of studied dandelion** microspecies: *Taraxacum balticum* (*T. bal*), *T. nordstedtii* (*T.nor I*, *T. nor II*) and *T. haematicum* (*T. hae*). Levels of similarity in leaf nutrient concentrations are indicated by a Euclidean’s distance.

The similarity analysis performed using Euclidean’s distances showed populations *T. haematicum* and *T. nordstedtii II* to be the closest to each other (Fig. 5); both populations were associated with non-saline and mostly mineral soils, poor in potassium, magnesium and calcium. These samples were characterized by low nutrient concentrations in the leaves. The most distinct position in the dendrogram was occupied by *Taraxacum balticum*. The population of this species was associated with the most saline soils, with the highest organic matter content, as well as K, Mg and Ca content. At the same time, the highest concentration of macro- and microelements was found in this species leaves.

## Discussion

The soil is very important factor for wild plant populations development. Some soil properties, e.g., pH, carbonates and available nutrients affect not only the growth and development of plants but also their chemical composition. Thompson et al. (1997) found leaf Ca and Mn concentrations affected by soil acidity, the correlations being positive and negative, respectively. For dicots, in contrast to monocots, those authors revealed also positive correlation for leaf Mg and negative correlation for leaf Zn. Similar results (significant negative correlations between soil pH and Mn, Zn, and Fe concentrations in leaves) were found by Roca-Pérez, Boluda & Pérez-Bermúdez (2004) in leaves of *Digitalis obscura*. On the other hand, the organic matter content was not correlated with micronutrient accumulation in leaves of the studied species (Roca-Pérez, Boluda & Pérez-Bermúdez, 2004). Our results are opposite to that. All correlations between macro- and microelement contents in the dandelion leaves and the soil properties studied (salinity, pH, organic matter, available forms of K, Mg, Ca) were positive and significant, with the exception of molybdenum concentration.

Similarities in leaf macro- and microelement concentrations between related taxa are considered to be the reflection partly of similarities of their biology and partly of the restriction to similar habitats (Thompson et al., 1997). Our results showed that, at least in specific habitats, the similarities in leaf nutrient concentrations between populations of separate microspecies occurring under similar habitat conditions are greater than similarities between populations of the same microspecies occurring in habitats with different conditions. Among all soil properties studied, soil salinity was the most important factor that differentiated populations of *Taraxacum* microspecies in terms of the mineral composition of the leaves.

Owing to the low salinity of the Baltic Sea, plants growing on Baltic coastal meadows are not, as a rule, exposed to a very strong salinity stress. The electrolytic conductivity of the saturated soil extract (ECe) at the sites located closest to the natural source of salinity did not exceed 4.5 dS m^−1^ (moderately saline soils); with the increasing distance from the shore, the conductivity was decreasing to 2.5 dS m^−1^ (slightly saline soils) down to a level typical of non-saline soils.

Differences in salinity stress effects on plants can result from the plant type (its tolerance strategies), salinity level and composition, micronutrient concentration, and growing conditions. The soil salinity can affect water uptake and nutrient availability (Grattan & Grieve, 1998; Parihar et al., 2015). Solubility of micronutrients (e.g., Cu, Fe, Mn, Mo and Zn) is usually low in saline soils, but not always. High concentrations of Na and Cl ions in a soil solution may depress ion activity and produce, e.g., extreme Na/Ca, Na/K, Ca/Mg ionic ratios. Potassium concentration usually declines as the Na-salinity is increased (Graifenberg et al., 1995; Keutgen & Pawelzik, 2009). Petropoulos et al. (2017) examined effects of salinity on, *inter alia*, the chemical composition of *Cichorium spinosum* cultivated at three salinity levels (2, 4 and 8 dS m^−1^). They found the salinity increase to reduce the K content and to increase the contents of Na, Ca, Mg, Mn, Fe and Zn. Our studies confirm this pattern, except for the potassium concentration being reduced as the soil salinity increased. The dandelion leaf K content in our study was positively correlated with the soil salinity, which was also the case in the study of Cachorro, Ortiz & Cerdá (1993) who showed that K may be transported against a strong Na concentration gradient, and that the K concentration in plants increased with increasing soil salinity.

The potassium content in leaves of the dandelions we examined was considerably higher than that of sodium, as is common in plants. Rácz-Kotilla, Rácz & Solomon (1974) showed a particularly high potassium content in *Herba taraxaci* that may be responsible for the plant’s strong diuretic effect. Compared to other studies on mineral composition of edible leafy plants, the main content of potassium in the leaves of all the dandelion microspecies we examined was lower than that in leaves of *Taraxacum officinale* (Escudero et al., 2003), *Cichorium intybus*, *Lactuca sativa* and *Eruca sativa* (Kawashima & Soares, 2003), whereas it exceeded the contents reported from leaves of *Cardaria draba* and *Crithmum maritimum* (Guil-Guerrero, Giménez-Martínez & Torija-Isasa, 1999) as well as *Sonchus oleraceus* and *Silybum marianum* (García-Herrera et al., 2014). Moreover, the mean potassium contents in leaves of *Taraxacum balticum* and *T. nordstedtii* (*I* and *II*) were higher than in *Taraxacum obovatum* and *Cichorium intybus* (García-Herrera et al., 2014); they were also higher in *Taraxacum balticum* than in *Lactuca sativa* (Kunachowicz et al., 2005) and *Chondrilla juncea* (García-Herrera et al., 2014). On the other hand, the mean contents of sodium in the leaves of the dandelion microspecies we examined were higher than in all the comparable edible leafy plants (Table 5). Guil-Guerrero, Giménez-Martínez & Isasa (1998) found the sodium content to be higher in halophytic edible plants (almost six times higher in the halophytic *Crithmum maritimium* than in the non-halophytic *Cardaria draba*). The K/Na ratios were also very variable, and ranged from 11.0 in *C. draba* to the minimum of 1.0 in *C. maritimium.* The K/Na ratios in our study were found to range within 1.2–1.5 in *Taraxacum balticum* and *T. nordstedtii I* growing on saline soils, and within 1.5–1.7 in *T. nordstedtii II* and *T. haematicum* growing on non-saline soils.

**Table 5:** Mean mineral component concentrations in different edible leafy plants (all the literature values converted to mg kg^−1^ dry weight).

Species	Na	K	Ca	Mg	Cu	Fe	Mn	Zn	Data source
*Taraxacum balticum*	1339.8	2014.3	505.6	168.5	1.8	15.4	2.9	7.5	present study
*Taraxacum nordstedtii I*	1065.2	1251.1	366.4	124.5	3.6	25.3	1.4	13.1	present study
*Taraxacum nordstedtii II*	466.9	1319.6	281.8	128.6	0.9	6.3	1.1	4.1	present study
*Taraxacum haematicum*	577.1	860.8	356.7	126.2	1.6	11.1	0.9	6.4	present study
*Taraxacum officinale*	–	2134.4	588.7	398.1	–	–	–	–	Escudero et al. (2003)
*Taraxacum obovatum*	58.3	945.2	195.4	30.4	0.2	5.9	0.5	0.8	García-Herrera et al. (2014)
*Chondrilla juncea*	61.0	1575.9	629.1	85.3	0.9	8.3	2.0	3.4	García-Herrera et al. (2014)
*Sonchus oleraceus*	177.0	677.3	193.5	39.8	0.1	1.0	0.5	0.7	García-Herrera et al. (2014)
*Silybum marianum*	53.5	473.9	87.1	11.4	0.1	0.3	0.1	0.2	García-Herrera et al. (2014)
*Cichorium intybus*	103.2	950.6	136.0	34.7	0.1	1.6	0.3	0.5	García-Herrera et al. (2014)
*Cichorium intybus*	190.0	6380.0	500.0	170.0	0.6	5.0	3.0	2.4	Kawashima & Soares (2003)
*Lactuca sativa*	50.0	3180.0	470.0	180.0	0.4	5.0	4.0	3.3	Kawashima & Soares (2003)
*Lactuca sativa*	40.0	1340.0	240.0	90.0	–	7.0	–	–	Kunachowicz et al. (2005)
*Eruca sativa*	40.0	3630.0	980.0	300.0	1.0	11.0	3.0	4.0	Kawashima & Soares (2003)
*Cardaria draba*	71.5	806.0	221.0	169.0	0.2	2.5	1.3	1.0	Guil-Guerrero, Giménez- Martínez & Torija-Isasa (1999)
*Crithmum maritimum*	377.0	403.0	126.1	106.6	0.2	3.0	0.9	0.6	Guil-Guerrero, Giménez- Martínez & Torija-Isasa (1999)

Studies on *Taraxacum obovatum* have shown it to be a rich source of calcium, particularly on account of its very low oxalic acid content (Guil et al., 1996; García-Herrera et al., 2014; Morales et al., 2014). This organic acid, commonly found in plants, inhibits Ca absorption through the formation of insoluble calcium oxalates. The lowest oxalic acid/calcium ratio found in the *T. obovatum* leaves suggests that Ca supplied by the plant would be more easily absorbed. Dandelion leaves, generally known for their low oxalic acid content, are recommended for consumption raw in salads for people who suffer from kidney calculus problems (Sánchez-Mata et al., 2012; Morales et al., 2014).

The mean content of calcium in leaves of all the dandelion microspecies we examined was lower than that in leaves of *Taraxacum officinale* (Escudero et al., 2003), *Chondrilla juncea* (García-Herrera et al., 2014) and *Eruca sativa* (Kawashima & Soares, 2003), whereas it was higher than in leaves of *Cardaria draba, Crithmum maritimum* (Guil-Guerrero, Giménez-Martínez & Torija-Isasa, 1999), *Lactuca sativa* (Kunachowicz et al., 2005), *Taraxacum obovatum, Cichorium intybus, Sonchus oleraceus* and *Silybum marianum* (García-Herrera et al., 2014) (Table 5).

The mean concentration of magnesium in leaves of the dandelion microspecies we examined was higher than that in leaves of *Crithmum maritimum* (Guil-Guerrero, Giménez-Martínez & Torija-Isasa, 1999), *Lactuca sativa* (Kunachowicz et al., 2005), *Taraxacum obovatum, Chondrilla juncea, Cichorium intybus, Sonchus oleraceus* and *Silybum marianum* (García-Herrera et al., 2014), and lower than in leaves of *Taraxacum officinale* (Escudero et al., 2003), *Cichorium intybus*, *Lactuca sativa* and *Eruca sativa* (Kawashima & Soares, 2003) (Table 5).

The calcium and magnesium contents in the dandelion leaves we examined was found to increase with increasing salinity, an effect similar to that reported by Petropoulos et al. (2017), but opposite to the results of studies described by Khan, Ungar & Showalter (2000), Tavakkoli et al. (2011) and Hussin, Geissler & Koyro (2013), who showed the Ca and Mg concentrations in plant organs to decline in response to the external NaCl salinity.

The mean concentrations of copper and zinc in leaves of all the dandelion species we examined was higher than those in all other comparable edible leafy plants. This was also the case with iron, except for *Taraxacum nordstedtii II* with the iron content lower than that in leaves of *Chondrilla juncea* (García-Herrera et al., 2014) and *Eruca sativa* (Kawashima & Soares, 2003). The mean concentration of manganese in the dandelion leaves we examined was higher than that in leaves of *Taraxacum obovatum, Cichorium intybus, Sonchus oleraceus, Silybum marianum* (García-Herrera et al., 2014) and *Crithmum maritimum* (Guil-Guerrero, Giménez-Martínez & Torija-Isasa, 1999); on the other hand, it was lower than that in leaves of *Cichorium intybus*, *Lactuca* sati*va* and *Eruca sativa* (Kawashima & Soares, 2003) (Table 5).

By examining the influence of environmental factors on properties of wild plants, we can detect, for example, the presence and concentrations of the desired/necessary elements in plants and recommend optimal sites and habitats for harvesting wild greens for commercial or personal use. Results of our studies on mineral composition of dandelion leaves lend support to the contention of, *inter alia*, Grivetti & Ogle (2000), that wild greens provide essential mineral nutrients to the diet and should be considered as a part of the total food system.

## Conclusions

Our results proved that dandelion microspecies differ significantly in the mineral composition of their leaves. The between-microspecies differences were significant for all the macroelements except magnesium and all the microelements except molybdenum. Soil conditions significantly affected nutrient concentrations in the dandelion leaves. We found positive and significant correlations between concentrations of most macro- and microelements in dandelion leaves and the soil properties, the leaf mineral contents being most strongly correlated with the soil salinity. Moreover, the differences between *Taraxacum nordstedtii I* and *T. nordstedtii II* populations occurring on saline and non-saline soils, respectively, turned out to be greater than between *T. nordstedtii II* and *T. haematicum* on non-saline soils and *T. nordstedtii I* and *T. balticum* on saline soils. This may mean that the impact of soil conditions on the macro- and microelement contents in plants is stronger than genetic differences between species, at least between those closely related.

Coastal meadows, fed by the brackish water of the Baltic Sea and free of anthropogenic pollution, are a good habitat to collect nutritionally valuable edible wild greens from. The plants growing on those meadows are not exposed to a very strong salinity stress and the resultant adverse effects for plant condition and properties.

##  Supplemental Information

10.7717/peerj.10233/supp-1 Supplemental Information 1 Raw data containing measurements of macro- and microelement concentrations in leaves of three *Taraxacum* microspecies as well as soil properties of localities from which the leaves were picked off

## Additional Information and Declarations

### Field Study Permissions

The following information was supplied relating to field study approvals (i.e., approving body and any reference numbers): In this area of study, plant management is allowed, including plant species harvesting. Taraxacum species studied are not protected by local law. In Poland, issues related to nature protection are regulated by the Nature Conservation Act of 16 April 2004 (Dz. U. z 2020 r. poz. 55). Article 36 of the said Act states that in the Natura 2000 area no type of activity is limited, including economic, agricultural, forestry, fishing, etc., as long as it does not adversely affect the conservation status of natural habitats. Coastal grasslands in NW Poland, within the Natura 2000 area PLH320018 are habitats that require regular human activity, including grazing and mowing. In Poland, only in two types of protected areas are there restrictions on free access, i.e. in national parks and nature reserves (Art. 15.1.24), but in our case, this article does not apply, because the research area is outside the area of any nature reserve or national park. For these reasons, no permission for conducting the research or collecting material was needed.

### Data Availability

The following information was supplied regarding data availability: The raw measurements are available in the Supplemental File.
